# Association of New York State’s Marketplace Special Enrollment Period for Pregnancy With Prenatal Insurance Coverage

**DOI:** 10.1001/jamahealthforum.2022.4907

**Published:** 2023-01-06

**Authors:** Erica L. Eliason, Maria W. Steenland

**Affiliations:** 1 Department of Health Services, Policy and Practice, Brown University School of Public Health, Providence, Rhode Island; 2 Population Studies and Training Center, Brown University, Providence, Rhode Island

## Abstract

This cross-sectional study uses Pregnancy Risk Assessment Monitoring System data to investigate the association between marketplace pregnancy special enrollment and prenatal insurance coverage in New York.

## Introduction

The Affordable Care Act established health insurance marketplaces in 2014 for private insurance plan enrollment. Federally required open enrollment is 2 to 3 consecutive months annually.^1^ Outside federal enrollment periods, state-run exchanges may offer special enrollment for qualifying events.^1^ Childbirth is a federal qualifying event, but before 2016, no state had included pregnancy.^2^ Without special enrollment, pregnant people must wait until open enrollment for marketplace coverage, which could delay insurance access.^2^ In 2016, New York became the first state to establish a pregnancy special enrollment period, later followed by 5 additional states and the District of Columbia.^2,3^ We investigated the association between establishing a pregnancy special enrollment period and prenatal insurance coverage in New York.

## Methods

In this cross-sectional study, we conducted a difference-in-difference analysis using Pregnancy Risk Assessment Monitoring System (PRAMS) data, comparing prenatal insurance coverage before (2014-2015) and after (2017-2019) New York’s policy change vs 17 control states with PRAMS data from 2014 to 2019. We omitted 2016 because the pregnancy period for most births in 2016 overlapped with prepolicy and postpolicy periods. This study followed the STROBE reporting guideline and was considered to be non–human participant research by the Brown University institutional review board.

We hypothesized that the benefits of the policy change would be greatest among pregnant people eligible for marketplace subsidies who had incomes above Medicaid eligibility levels. We restricted the sample to respondents above New York’s pregnancy-related Medicaid eligibility (223% of the federal poverty level) and below 400% of the federal poverty level. The outcomes were prenatal marketplace coverage; employer, private, or military coverage; Medicaid; uninsurance; or other coverage.

We calculated weighted respondent characteristics in New York and control states, including self-reported race and ethnicity from birth certificates. Regression models included an interaction term between birth in New York and postpolicy births. Models included state and year fixed effects, with SEs clustered by state. Multivariable models were adjusted for demographic factors and previous live births. Analyses were weighted to account for the complex survey design. Methods for evaluating model assumptions, categorizing insurance types, and identifying which PRAMS income categories met inclusion criteria are described in eMethods 1 to 3 in the Supplement. Analyses were conducted between April 7 and September 8, 2022, using Stata, version 17 software (StataCorp LLC).

## Results

The study included 13 753 births (1167 in New York, 12 586 in control states), representing a weighted total of 712 175 births. Relative to controls (n = 4014), New York respondents (n = 354) were older (20.1% aged 35-39 years vs 14.0%) and less likely to be married (Table 1). In New York, unadjusted estimates of prenatal marketplace coverage significantly increased from 3.3% to 8.6% after the pregnancy special enrollment policy, while uninsurance fell from 1.5% to 0.6%. In control states, there were significant decreases in unadjusted estimates of other prenatal private or military coverage and increases in prenatal Medicaid from prepolicy to postpolicy. In adjusted models, New York’s pregnancy special enrollment period was associated with an increase of 6.3 (95% CI, 4.4-8.2) percentage points in the prenatal marketplace and decreases of 1.4 (95% CI, −2.1 to −0.7) percentage points in uninsurance and 3.0 (95% CI, −3.8 to −2.3) percentage points in other coverage relative to control states (Table 2). The policy was not associated with significant changes in other private, military, or Medicaid coverage.

**Table 1.** Prepolicy Demographic Characteristics of the Study Sample,^a^ Pregnancy Risk Assessment Monitoring System (PRAMS) 2014-2015

Characteristic	New York		Control states	
	Unweighted, No.	Weighted, % (95% CI)	Unweighted, No.	Weighted, % (95% CI)
No.	354	32 532	4014	191 597
Age, y				
≤24	38	12.6 (8.8-17.8)	626	15.0 (13.5-16.6)
25-29	115	33.4 (27.5-39.7)	1459	37.8 (35.7-40.0)
30-34	108	29.7 (24.1-36.0)	1292	30.7 (28.7-32.7)
35-39	74	20.1 (15.3-25.9)	538	14.0 (12.6-15.6)
≥40	19	4.2 (2.4-7.3)	99	2.5 (1.9-3.3)
Race and ethnicity^b^				
Black	64	13.5 (10.1-17.9)	429	8.0 (7.0-9.2)
Hispanic	73	20.4 (15.7-26.0)	393	8.7 (7.8-9.7)
White	170	54.0 (48.2-59.8)	2655	74.5 (72.9-76.0)
Other^c^	47	12.0 (8.6-16.6)	524	8.5 (7.6-9.5)
Education				
High school or less	58	16.4 (12.0-22.1)	725	17.9 (16.2-19.7)
More than high school	296	83.6 (77.9-88.0)	3248	81.5 (79.7-83.1)
Marital status				
Married	237	68.3 (61.8-74.1)	3103	77.0 (75.0-78.8)
Not married	117	31.7 (25.9-38.2)	909	23.0 (21.1-25.0)
Survey language				
English	339	95.6 (92.3-97.6)	3940	98.2 (97.5-98.7)
Spanish or Chinese	15	4.4 (2.4-7.7)	74	1.8 (1.3-2.5)
No. of previous live births				
0	171	43.4 (37.1-49.9)	1679	39.5 (37.4-41.6)
1	128	38.7 (32.6-45.3)	1582	41.3 (39.2-43.5)
2	42	12.8 (8.9-18.0)	586	15.5 (14.0-17.2)
≥3	12	4.5 (2.3-8.5)	155	3.5 (2.8-4.3)

^a^ N = 4368; weighted N = 224 129. Unweighted sample sizes and weighted proportions are presented using PRAMS survey weights. Sample with full income ranges from 223% to 400% of the federal poverty level in New York (New York City and New York State) and 17 control group states (Alaska, Delaware, Iowa, Illinois, Massachusetts, Maryland, Maine, Missouri, New Hampshire, New Jersey, New Mexico, Pennsylvania, Rhode Island, Utah, Washington, Wisconsin, and Wyoming). Spanish and Chinese were combined for survey language categories because PRAMS surveys were only available in Chinese in New York.

^b^ Race and ethnicity were self-reported in the birth certificate files.

^c^ Other categories were Alaska Native, American Indian, Asian, Pacific Islander, mixed race, and other race and ethnicity.

**Table 2.** Estimates of the Association Between New York State’s Marketplace Special Enrollment Period for Pregnancy and Prenatal Insurance Coverage, Pregnancy Risk Assessment Monitoring System (PRAMS) 2014-2019^a^

Prenatal coverage	% (95% CI)						Difference in differences, percentage points (95% CI)	
	New York (n = 1167; weighted n = 108 640)			Control states (n = 12 586; weighted n = 603 535)				
	Prepolicy	Postpolicy	Difference	Prepolicy	Postpolicy	Difference	Unadjusted	Adjusted
Marketplace	3.3 (1.5 to 7.1)	8.6 (6.0 to 12.3)	5.3 (1.3 to 9.3)^b^	6.2 (5.2 to 7.3)	5.3 (4.6 to 6.1)	−0.9 (−2.2 to 0.4)	6.2 (4.4 to 8.0)^c^	6.3 (4.4 to 8.2)^c^
Employer, other private, or military	70.3 (64.0 to 75.9)	64.4 (59.2 to 69.2)	−5.9 (−13.7 to 1.9)	79.5 (77.7 to 81.3)	76.2 (74.6 to 77.7)	−3.3 (−5.7 to −1.0)^d^	−2.6 (−5.3 to 0.1)	−1.2 (−3.9 to 1.6)
Medicaid	18.6 (14.0 to 24.3)	20.2 (16.4 to 24.7)	1.6 (−5.0 to 8.2)	10.6 (9.2 to 12.0)	12.5 (11.3 to 13.7)	1.9 (0.1 to 3.8)^b^	−0.2 (−1.9 to 1.4)	−1.4 (−3.0 to 0.2)
Uninsured	1.5 (0.6 to 3.5)	0.6 (0.1 to 4.4)	−0.8 (−2.6 to 0.9)	1.1 (0.8 to 1.6)	1.5 (1.1 to 2.1)	0.4 (−0.3 to 1.0)	−1.2 (−1.9 to −0.6)^d^	−1.4 (−2.1 to −0.7)^c^
Other	5.5 (3.2 to 9.4)	3.0 (1.7 to 5.3)	−2.6 (−6.0 to 0.9)	1.6 (1.2 to 2.2)	2.0 (1.6 to 2.5)	0.4 (−0.3 to 1.1)	−2.9 (−3.6 to −2.2)^c^	−3.0 (−3.8 to −2.3)^c^

^a^ N = 13 753; weighted N = 712 175. Weighted proportions are presented using PRAMS survey weights. Sample with full income ranges from 223% to 400% federal poverty level in New York (New York City and New York State) and 17 control group states (Alaska, Delaware, Iowa, Illinois, Massachusetts, Maryland, Maine, Missouri, New Hampshire, New Jersey, New Mexico, Pennsylvania, Rhode Island, Utah, Washington, Wisconsin, and Wyoming). Marketplace coverage includes insurance from the health care exchange or purchased directly from the insurance company. Employer or parental private or military coverage includes private insurance through work or through a parent, TRICARE, or other military insurance. Uninsurance includes individuals with Indian Health Service coverage. Adjusted difference-in-difference model includes age, educational attainment, marital status, language of survey completion, number of previous live births, and state and year fixed effects. Spanish and Chinese were combined for survey language categories because PRAMS surveys were only available in Chinese in New York. 2016 Omitted as a transition year.

^b^ Two-sided *P* < .05.

^c^ Two-sided *P* < .001.

^d^ Two-sided *P* < .01.

## Discussion

New York’s pregnancy special enrollment period was associated with increased marketplace coverage and decreased uninsurance among eligible pregnant people. Previous research has shown that gaps in marketplace coverage are common for eligible and enrolled pregnant people and associated with reduced prenatal care receipt.^4,5^ Study limitations include that coverage was measured as prenatal care payer, which did not capture when coverage started or duration of uninsurance. This analysis can inform policy debates about how to improve pregnancy insurance coverage, care, and health outcomes in the 18 states that can establish their own exchanges’ enrollment periods.^3,6^

## Notes

Supplement 1. **eMethods 1.** Difference-in-Difference Model Assumptions **eMethods 2.** Sample Selection **eMethods 3.** Definition of Outcomes

Supplement 2.
Data Sharing Statement
