# Supplementary material for: Association of New York State’s Marketplace Special Enrollment Period for Pregnancy With Prenatal Insurance Coverage
Source: JAMA Health Forum. 2023 Jan 6;4(1):e224907. doi: 10.1001/jamahealthforum.2022.4907 (PMC9857356; doi:10.1001/jamahealthforum.2022.4907)
Supplement: Supplement 1. — eMethods 1. Difference-in-Difference Model Assumptions eMethods 2. Sample Selection eMethods 3. Definition of Outcomes [file jamahealthforum-e224907-s001.pdf]

Supplemental Online Content

Eliason EL, Steenland MW. Association of New York State's marketplace special enrollment period for pregnancy with prenatal insurance coverage *JAMA Health Forum*. 2023;4(1):e224907. doi:10.101/jamahealthforum.2022.4907

eMethods 1. Difference-in-Difference Model Assumptions

eMethods 2. Sample Selection

eMethods 3. Definition of Outcomes

This supplemental material has been provided by the authors to give readers additional information about their work.

© 2023 Eliason EL et al. *JAMA Health Forum.*

eMethods 1. Difference-in-Difference Model Assumptions

We examined trends in the pre-policy period in New York and control group states in two ways. First, through linear pre-trend tests and second through event study models.

1) The description of the linear pre-trend test is below:

$y_{ity} = \beta 0 + \beta 1 Treated_t{*}LinearTimeTrend_y + \beta 2 Year_y + (YearFE)_y + (StateFE)_t + \gamma`x_{ity} + \varepsilon_{ity}$

y_{ity} is the outcome for an individual i in treatment group t in year y. β1 is an interaction term between treated group status and a linear time trend, assessing if there were differential linear trends in outcomes in the pre-policy period prior to 2016 in New York (treated) and control group states. Year indicates the year of birth in PRAMS. YearFE is the set of year fixed effects and StateFE is the set of state fixed effects. $\gamma`x_{it}$ is a vector of control variables, including respondent age, educational attainment, marital status, language of survey completion, and number of previous live births. For language of survey completion, Spanish and Chinese were combined for survey language categories because PRAMS surveys were only available in Chinese in New York. We are limited to only two pre-policy years for the pre-trends analysis as Marketplaces were established in 2014 and the New York policy occurred in 2016. There were no statistically significant differences in pre-trends in the study outcomes comparing NY with control states.

2) The description for the event study models is below:

The equation for the event study model follows the equation for the difference-in-difference model, replacing the interaction term between an indicator for birth in NY and an indicator for post-policy births with an interaction between the indicator for birth in NY and indicators for each year 2014-2019 relative to 2015, the year just prior to New York's 2016 policy change. These interaction term coefficients represent the differences in prenatal insurance coverage in New York compared to control states for each year relative to the difference in the omitted year (2015). For parallel trends in the pre-policy period, interaction term coefficients prior to 2015 should be close to zero and not statistically significant. Estimates are adjusted for age, educational attainment, marital status, language of survey completion, number of previous live births, and state and year fixed effects. We find no evidence of statistically significant differences in trends in the pre-policy period in event study models.

© 2023 Eliason EL et al. *JAMA Health Forum.*

eMethods 2. Sample Selection

Marketplace subsidies are available for individuals with incomes above 138% of the federal poverty level (FPL) up to incomes 400% of the FPL in expansion states. As a result, we limited the sample to Marketplace-eligible individuals with incomes below 400% of the FPL. We hypothesized that effects of the policy would be greatest among respondents who had incomes above Medicaid eligibility levels for pregnant people through pregnancy-related Medicaid. Therefore, we restricted the sample to respondents above New York's pregnancy-related Medicaid eligibility level, 223% of the FPL. During this time, the average pregnancy-related Medicaid eligibility level in control states was 214% of the FPL, so respondents in both New York and control states with incomes 223%-400% of the FPL would be both eligible for Marketplace subsidies and above the income eligibility threshold for pregnancy-related Medicaid.

In PRAMS, the household income measure is categorical. Respondents were considered to have incomes 223-400% of the FPL if their incomes fell into this range based on both the minimum and maximum values of the categorical income measure for their household size. For example, a 2019 respondent with a household size of 3 reporting income in the $48,001-$57,000 category would be included as their lowest potential FPL would be 225% and their highest potential FPL would be 267% based on this income range. Reflecting Office of Economic Opportunity practices, Alaska has separate poverty guidelines to determine financial eligibility from the lower 48 contiguous states. As a result, we used these separate poverty guidelines for calculating FPL in Alaska for all years.

New York City collects PRAMS data separately from the rest of New York State. We included both New York City and New York State to comprise the treated group for New York. For control group states, we included all 17 states that were consistently in PRAMS throughout the study period: Alaska, Delaware, Iowa, Illinois, Massachusetts, Maryland, Maine, Missouri, New Hampshire, New Jersey, New Mexico, Pennsylvania, Rhode Island, Utah, Washington, Wisconsin, and Wyoming.

© 2023 Eliason EL et al. *JAMA Health Forum.*

eMethods 3. Definition of Outcomes

The study outcome was type of prenatal health insurance coverage categorized into five groups. Prenatal coverage was determined by self-reported response to the PRAMS question: "During your most recent pregnancy, what kind of health insurance did you have for your prenatal care?" Prenatal insurance coverage types were categorized as: prenatal Marketplace coverage, employer/parental private or military coverage, Medicaid, uninsurance, or other coverage.

Outcomes	PRAMS Insurance Coverage Categories
Prenatal Marketplace	Insurance paid by State Health Insurance Marketplace, HealthCare.gov, or purchased directly from insurance company
Prenatal Employer, Other Private, or Military	Insurance paid by job, job of husband or partner, parents, TRICARE or other military health care
Prenatal Medicaid	Insurance paid by Medicaid
Prenatal Uninsurance	Did not have any health insurance to pay for prenatal care or Indian Health Service coverage only
Prenatal Other Coverage	Insurance paid by SCHIP/CHIP, other government, or other

© 2023 Eliason EL et al. *JAMA Health Forum.*
