# Supplementary material for: Association of New York State’s Marketplace Special Enrollment Period for Pregnancy With Prenatal Insurance Coverage
Source: JAMA Health Forum. 2023 Jan 6;4(1):e224907. doi: 10.1001/jamahealthforum.2022.4907 (PMC9857356; doi:10.1001/jamahealthforum.2022.4907)
Supplement: Supplement 2. — Data Sharing Statement [file jamahealthforum-e224907-s002.pdf]

Data Sharing Statement

Eliason. Association of New York State's Marketplace Special Enrollment Period for Pregnancy With Prenatal Insurance Coverage. *JAMA Health Forum*. Published January 06, 2023. doi:10.1001/jamahealthforum.2022.4907

Data
Data available: No
